# Analysis on Target Detection and Classification in LTE Based Passive Forward Scattering Radar

**DOI:** 10.3390/s16101607

**Published:** 2016-09-29

**Authors:** Raja Syamsul Azmir Raja Abdullah, Noor Hafizah Abdul Aziz, Nur Emileen Abdul Rashid, Asem Ahmad Salah, Fazirulhisyam Hashim

**Affiliations:** 1 Wireless and Photonic Networks Research Centre, Faculty of Engineering, Universiti Putra Malaysia (UPM), Serdang 43400, Malaysia; asemsalah@gmail.com (A.A.S.); fazirul@upm.edu.my (F.H.); 2 Faculty of Electrical Engineering, Universiti Teknologi MARA (UiTM), Shah Alam 40450, Malaysia; noor4083@salam.uitm.edu.my (N.H.A.A.); emileen98@salam.uitm.edu.my (N.E.A.R.)

**Keywords:** passive forward scattering radar sensor, target detection, target classification

## Abstract

The passive bistatic radar (PBR) system can utilize the illuminator of opportunity to enhance radar capability. By utilizing the forward scattering technique and procedure into the specific mode of PBR can provide an improvement in target detection and classification. The system is known as passive Forward Scattering Radar (FSR). The passive FSR system can exploit the peculiar advantage of the enhancement in forward scatter radar cross section (FSRCS) for target detection. Thus, the aim of this paper is to show the feasibility of passive FSR for moving target detection and classification by experimental analysis and results. The signal source is coming from the latest technology of 4G Long-Term Evolution (LTE) base station. A detailed explanation on the passive FSR receiver circuit, the detection scheme and the classification algorithm are given. In addition, the proposed passive FSR circuit employs the self-mixing technique at the receiver; hence the synchronization signal from the transmitter is not required. The experimental results confirm the passive FSR system’s capability for ground target detection and classification. Furthermore, this paper illustrates the first classification result in the passive FSR system. The great potential in the passive FSR system provides a new research area in passive radar that can be used for diverse remote monitoring applications.

## 1. Introduction

The advantages of Forward Scattering Radar (FSR) have created a ‘comeback’ interest in the system in the past decade. This is followed by active research throughout the world from Universities, Research Institutes and Industries [1,2,3,4]. The main difference between the FSR and the traditional radar is that the operational mode of the FSR corresponds to the case where the bistatic angle is near 180°. The key difference of the FSR in contrast to its monostatic and bistatic counterparts is that it utilizes the effect of electromagnetic waves, shadowing a target rather than reflecting from the target. The FSR system exhibits some special characteristics such as: enhanced target radar cross sections, not effected by stealth techniques for radar signal reduction, long coherent intervals of the receiving signal, absence of signal fluctuations and simple hardware [1,2].

In other important areas of the radar system, passive radar has become very crucial and much research has been published comprising theoretical and practical applications with rapid developments. The passive radar system is made up of a receiver without the co-located transmitter. Hence, one factor that makes passive radar interesting is the possibility of utilizing an illuminator of opportunity, which can reduce the cost of a radar system due to the no expenses needed to be paid for a transmitter system. The basis of not having a dedicated transmitter leads to the system being practically invisible to surveillance. To date, many illuminators of opportunity have been employed by many sources such as: Television Broadcasting [5], FM radio [6], Digital Video and Audio Broadcasting (DAB) [7], satellites [8,9], Global Systems for Mobile communications (GSM) [10] and Long Term Evolution (LTE) [11].

The receiver circuit for conventional PBR using the heterodyne concept requires a reference signal from direct transmission for synchronization. The signal is then down converted to the base band. Target detection is evaluated by analyzing the ambiguity function of the received signal. Alternatively, this paper proves that by having hybrid operation between the FSR technique and conventional passive radar is beneficial due to: (i) a simple FS receiver circuit; (ii) significant improvement in radar cross section (RCS) at the forward scatter main lobe (FSML); (iii) passive FSR does not require direct signal from the base station and (iv) target detection scheme based on signal envelope is straight forward without complicated signal pre-processing step. Furthermore, the classification capability in passive FSR increases the effectiveness of the system [1,12]. The interesting feature in passive FSR, which is the sharp rise of the FS RCS, can increase the sensitivity of the system, making it robust to “Stealth” technology. This effect which exists within the FSML can only be seen within a specific condition in the Mie region and the optical region [13].

Several researches have been carried out studying the feasibility of using passive radar with the FSR mode. In the meantime, studies on using GPS signals as a passive radar system have been demonstrated in [14,15] by using GPS L1 and GPS L5 respectively for air target detection. Passive radar to detect ground targets by using the GPS signal is explained in [16] with positive experimental results. Despite the possibility of using GPS as passive radar, however, the proposed radar system is limited to the signal availability due to satellite movement, signal blockage by buildings or trees and difficulty in tracking the satellite position. A research using GSM as a signal source and utilizing the forward scatter geometry for target detection is explained in [17]. However, the proposed system deployed a conventional passive radar receiver mechanism by using two antennas; one for reference and the other one to receive signals from a target. Recent publications on passive forward scatter radar have introduced a new algorithm for signal detection, which is for air target detection. For air target, experimental results illustrated that it could detect an airplane crossing the FSR baseline by using DVB-T signals [3].

Despite the research done so far, the literature is lacking in the results for ground target detection and classification in passive FSR, especially the experimental investigation. Thus, the aim of this paper is to show the feasibility of passive FSR for moving target detection and classification by experimental analysis and results. In addition, the target’s distance from the radar sensor can also be determined. The analysis was carried out by exploiting the actual LTE signal transmitted through the air from the base station. Three different vehicles that represent three different categories were used during the test campaign. The three vehicle categories are named as: Compact, Saloon and Small Sport Utility Vehicle (SUV). The passive FSR experimental results highlight two important findings. First, the vehicles were successfully detected, even by raw received signals without any complicated signal processing techniques. Second, the proposed classification system provides results of excellent classification performance. The results from this paper can enhance the passive radar system capability by integrating the FSR mode onto the conventional PBR system.

The LTE has remarkable characteristics that make it attractive to be used as an illuminator of opportunity for passive radar applications. Among the characteristics, the LTE signal has a broad bandwidth differs from 1.4 MHz to 20 MHz which provides a high range resolution in comparison to other illuminators of opportunity (i.e., GSM, DVB and DAB). The variety of the LTE allocated frequency bands (800 MHz–3.5 GHz) allowing the LTE to be deployed in many countries, consequences a future broad coverage of the LTE in the worldwide [18]. Additionally, the number of commercial LTE networks is seeing a massive increase year by year. Therefore, increasing the LTE signal availability will expand LTE-based passive radars’ deployment opportunities. Analysis on the LTE signal Ambiguity Function (AF) shows that low side-lobes and high range resolution can be achieved [19]. The above-mentioned LTE signal characteristics and others motivated the authors to use the LTE signal as an illuminator of opportunity for passive FSR system.

The layout of the paper is organized as follows: Section 2 focuses on the target detection technique in passive FSR by exploiting the enhancement of the target RCS. Moreover, the fundamental received signal equation is derived and the proposed passive FSR system is also explained. Section 3 elaborates on the approach for target classification by using the PCA in the first part, followed by target range discrimination and classification performance analysis. The conclusion and direction for future work are outlined in the last section.

## 2. Target Detection in Passive Forward Scattering Radar

Radar cross section (RCS) information and pattern is one of the parameters in target’s detection process and in feature’s extraction for classification techniques of the proposed passive FSR system. The second parameter is the target’s Doppler frequency in the received signal due to relative movement between the target and radar. This section will explain the target detection method and practical experimental set up for a passive FSR system.

### 2.1. Passive Forward Scatter Radar Cross Section

**Figure 1:**
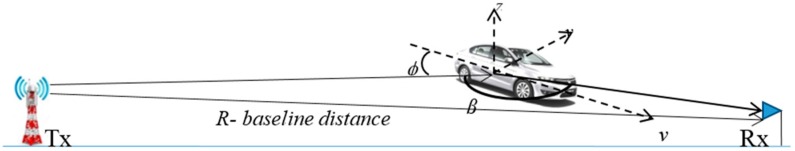
Topology of passive forward scatter radar setup showing wave incident angle, *φ*.

**Figure 2:**
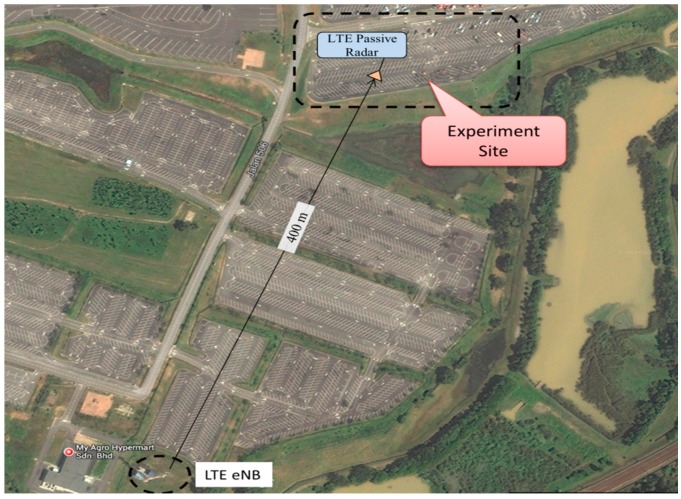
Aerial photo of geometrical experimental site showing LTE base station.

The target’s RCS of a real model was first estimated by using Computer Simulation Technology (CST) Microwave studio software for different vehicle shapes, sizes and also plane wave angles of incident, *φ*. In theory, the RCS depends on the target’s physical geometry and exterior features, the direction of the illuminating radar, the bistatic angle, *β*, the radar transmitter frequency and the types of material used. Two wave incident angles (*φ* = 59° and *φ* = 90°) were analyzed, which follows the experimental setup and geometrical scenario as illustrated in Figure 1 and Figure 2 respectively.

**Figure 3:**
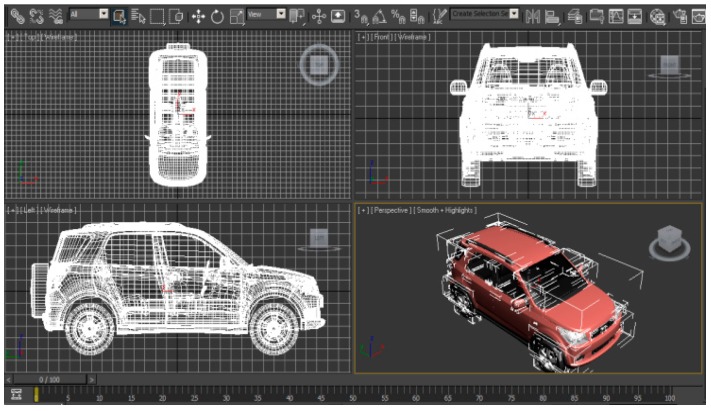
Model of a Small SUV for RCS calculation.

**Figure 4:**
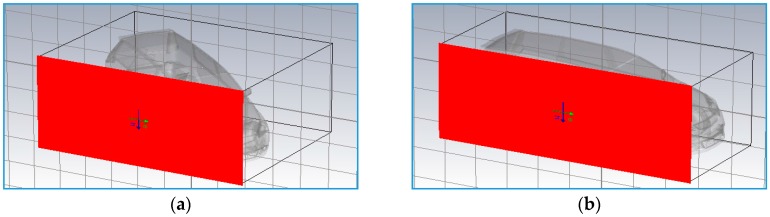
Radar source towards target at incident angle, *φ* at (**a**) 59° and (**b**) 90°.

The Cartesian and polar plots of vehicle’s RCS were analyzed in order to examine the effect of incident angle to the RCS in passive FSR. The models of the vehicles were designed in Autodesk software based on the actual dimensions of the vehicles. As an example, in this paper, the dimension of the Small SUV is L = 4420 mm, W = 1695 mm, H = 1740 mm as shown in Figure 3. These models were then exported to the CST for simulation and the plane wave from LTE base station is used as the radar signal’s source. The RCS was calculated for the 2.6 GHz LTE carrier frequency and angles of incident plane wave, *φ* at 59° and 90° as shown in Figure 4.

**Figure 5:**
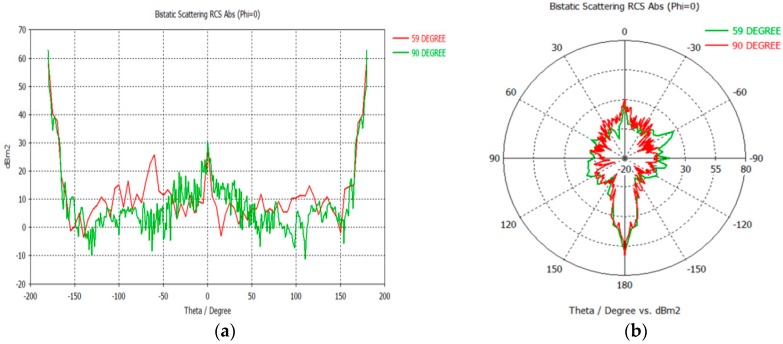
Vehicles’ RCS at two different incident angles: (**a**) Cartesian Plot and (**b**) Polar Plot.

The influence of the cross sectional shape and the size of the vehicles on the RCS are presented in the RCS radiation pattern for all bistatic angles at the LTE carrier frequency of 2.6 GHz. The results were analyzed in the form of polar and Cartesian plots as shown in Figure 5. The RCS attributes for the Small SUV at two incident angles are compared in Table 1. The figure and table point out that the amplitude of RCS when *φ* = 90° is higher than *φ* = 59°, and can be seen at the main lobe magnitude. Moreover, this result explains that the vehicles more likely to be detected at an incident angle, *φ* = 90°. The side lobe level of *φ* = 90° is smaller compared to *φ* = 59°, which highly fluctuated. This could be the fact that the target’s silhouette of the vehicle is much more visible at *φ* = 90°. However, regardless of the angle of the incident wave, the RCS is always maximum at FSR (*β* = 180°). This is the main advantage of using forward scattering mode in passive radar where the effects on enhanced RCS can be seen [20].

### 2.2. Target Doppler Signature in Passive FSR

**Figure 6:**
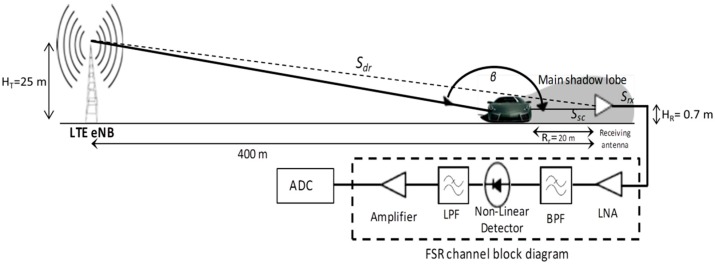
Passive Forward Scattering Radar sensor system based on LTE signal transmission.

To analyze the feasibility of using LTE signal for passive FSR, an outdoor test with actual LTE signal transmission has been carried out. The aim of the experiment is to capture signal scattered from a moving vehicle crossing the transmitter and receiver baseline in forward scatter mode prior to the classification system. Figure 6 illustrates the outdoor testing scenario and block diagram of the hardware used for the system. Unlike conventional passive radar systems, only one channel is required facing the LTE base station in the proposed passive FSR. This is due to the receiver applies a self-mixing technique by the non-linear element, thus it does not require any synchronization channel. Overall, the receiver system was made of low noise amplifier (LNA), a diode (a non-linear element as amplitude detector), a low pass filter (LPF), a low frequency amplifier and an ADC. When a moving target entering the FS mode, two signals were scattered to the FS receiving antenna; a direct signal from the LTE base station transmission, *S_dr_*, (assume phase noise free) and a scattered signal from a moving target, *S_sc_* carrying a Doppler shift, *ω_sc_* [2,13] as depicted in Equations (1) and (2) respectively. In a simplified manner, the received signal at the input of the passive FSR system *S_rx_* can be expressed as Equation (3):

(1) $$S_{dr}\left(t\right)=A_{dr}\cos\left(\left(\omega_{o}\right)t\right)$$

(2) $$S_{sc}\left(t\right)=A_{sc}\sin\left(\left(\omega_{o}+\omega_{sc}\right)t\right)$$

$$S_{rx}\left(t\right)=S_{dr}\left(t\right)+S_{sc}\left(t\right)$$

(3) $$S_{rx}\left(t\right)=A_{dr}\cos\left(\left(\omega_{o}\right)t\right)+A_{sc}\sin\left(\left(\omega_{o}+\omega_{sc}\right)t\right)$$

where *A_dr_* represents the amplitudes of the direct transmission signal (leakage) and *A_sc_* is the amplitude of the signal scattered from the moving target. As stated earlier, the system uses a non-linear element as its detection mechanism with the transfer characteristics of *Y_out_* = *(Y_in_)*^2^, hence at the output of the non-linear element and in this case the self-mixing between *S_dr_* and *S_sc_*, the signal could be represented by:

(4) $$Y_{out}\left(t\right)={\left(A_{dr}\cos\left(\omega_{o}t\right)+A_{sc}\sin\left(\left(\omega_{o}+\omega_{sc}\right)t\right)\right)}^{2}$$

The LPF is then filtered out the high frequency part at the output signal from the non-linear element, so only two spectral components that contained in *Y_out_*; the direct path signal (leakage signal) and the signal with Doppler frequency scattered from the target. The filtered signal is denoted as *Y_out_f_* and shown in Equation (5):

(5) $$Y_{out\_f}\left(t\right)=Κ\left(\left({\left(A_{dr}\right)}^{2}+{\left(A_{sc}\right)}^{2}\right)+\left(A_{dr}A_{sc}\sin\left(\omega_{sc}t\right)\right)\right)$$

whereby, K is the conversion coefficient that depends on the type of non-linear device used. The direct signal in Equation (5) can be filtered out by using HPF (the system can use either hardware or software filter), it will then retain only the Doppler component as *Y_out_f_ = A_dr_A_sc_sin(ω_sc_t)*. The total Doppler frequency can be extracted by deriving the phase component of Equation (5): and is given by:

(6) $$f_{d}=\frac{2v}{\lambda }\sin\alpha$$

Equation (6) indicates the Doppler shift depends on the target’s velocity vector components (in the direction to the receiver) and the carrier frequency, λ. The resulted Doppler shift can be used for moving target detection, speed determination and trajectory reconstruction [2]. The Doppler component of the received waveform also contains information about the target silhouette. In this paper, this information was used for vehicle classification and recognition. The target Doppler frequency can be calculated as below. The target Doppler frequency component due to motion relative to the transmitter and receiver is given in Equations (7) and (8) respectively:

(7) $$f_{tx}=\left(v/\lambda \right)cos\left(\beta /2+\delta \right)$$

(8) $$f_{rx}=\left(v/\lambda \right)cos\left(\beta /2-\delta \right)$$

whereby, *δ* = 180° − *β/2*, and the total target Doppler frequency:

$$f_{d}=f_{tx}+f_{rx}$$

$$f_{d}=\left(v/\lambda \right)[\cos\left(\beta /2+\delta \right)+\cos\left(\beta /2-\delta \right)]$$

(9) $$f_{d}=\left(2v/\lambda \right)\cos(\beta /2)\cos(\delta )$$

Based on Equation (9), the maximum Doppler shift occurred when the target trajectory perpendicular to the baseline (*δ* = 0°) and therefore, the target frequency Doppler is determined by target’s speed. The higher the target’s effective speed, the higher the Doppler shift at the received signal.

### 2.3. Received Signal and Target’s Features in Passive FSR

**Figure 7:**
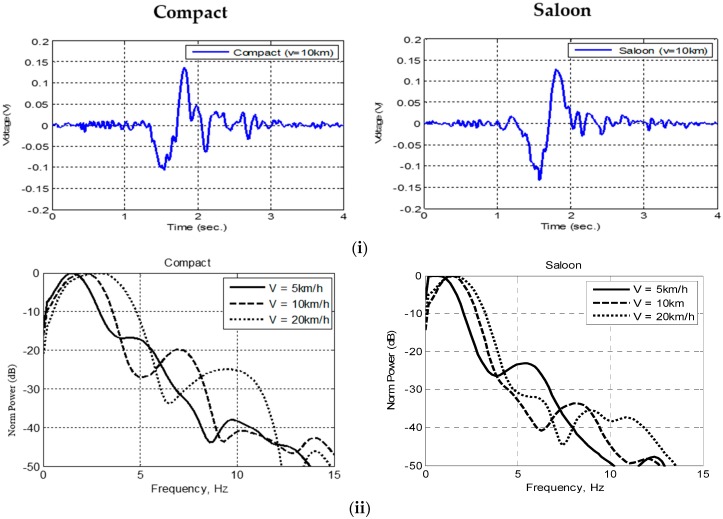

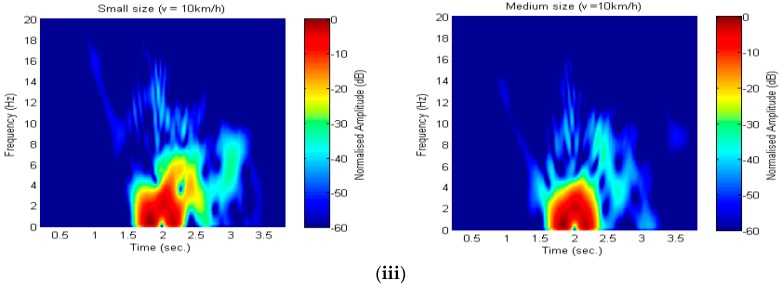
Received scattered signal in (**i**) time domain; (**ii**) power spectrum and (**iii**) spectrogram of moving vehicles crossing the Tx-Rx radar baseline for Compact and Saloon.

In FSR system, high Doppler resolution can be achieved by long received signal coherent integration time [1,2]. The coherent integration time is determined by the duration of target’s visibility within the FSR signal beam width. Moreover, the target’s relative speed, which is beyond radar control, is an important parameter in passive FSR. Based on previous work, in practice, information on the target speed can be obtained from the received signal, as explained by Equation (9) and investigated in the next section. The experimental time domain signal scattered from twodifferent cars: Compact and Saloon are shown in Figure 7i respectively. The envelope of the received signal’s amplitude in time domain is used for vehicle detection in passive FSR. The signal also shows that the horizontal (time) scale of the waveform proportional to the vehicle’s speed. Both signals showed the target crossing the baseline with an almost symmetrical shape for the Doppler frequency. Fundamentally, the obtained results show two significant relationships:

- the amplitudes of Doppler signatures to the target’s forward scatter cross section, hence, the Saloon car had a bigger forward scatter cross section, which was reflected in the amplitudes of their signature,
- the signal span in time domain to the length of the target, hence, the Saloon car also had a longer body, which was reflected in the signal span of the forward scatter main lobe.

In addition, the Doppler modulated in the signal can be clearly seen at both ends of the signals when the car was entering and leaving the baseline, and when the antenna started to receive the target’s forward scatter lobe.

The signal’s power spectra densities of the same cars at different speeds are shown in Figure 7ii. From the FSR theory, the position of the first null can be approximately determined from the ratio between the speed and the length of the vehicle [2]. This relationship can be used to estimate the vehicle’s speed. The Doppler spectrum signature is unique for specific vehicle’s shapes and dimensions. It is used as the vehicle’s feature vector for classification system in this paper. Several meaningful criteria can be analyzed and selected as features, for example, the number of spectrum lobes, the number of principal components to be used and minimum power level.

Figure 7iii shows the signal spectrograms for each vehicle. Two main characteristics based on the spectrograms can be point out: the size of the vehicle’s forward scatter main lobe can be estimated, and it also illustrates that each vehicle has unique time-frequency attributes and features that can be utilized for advanced vehicle recognition.

## 3. Target Classification in Passive Forward Scattering Radar

The different categories of vehicle used for the classification is shown in Table 2. This paper chose three vehicle categories to be analyzed. These categories are assumed to be the most suited representation of the typical vehicle’s type on the road. In addition, if the system can classify these three vehicle categories which have close proximity in size between them, it could then be much easier to classify for vehicle with huge different in size between them (for example lorry and motorbike). During all the testing campaign, the same vehicles as in Table 2 were used to represent the type of category, even at different day and time of experimental campaigns.

**Figure 8:**
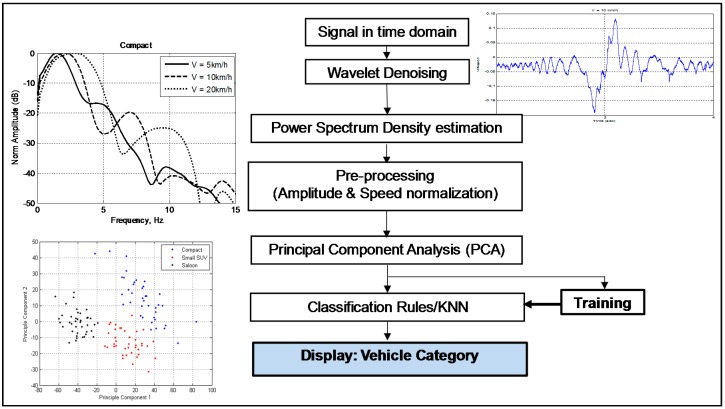
Classification system block diagram in passive FSR using LTE signal.

Figure 8 illustrates the classification block diagram used in the proposed passive FSR system. It is divided into two sub-systems: the signal pre-processing that analyzes the input time-domain signature including signal normalization. The second sub-system consists of the classification itself, which includes features extraction, training and testing. In the pre-processing block, the time domain signature is first de-noised using wavelet to smooth the signal. Then, the Power Spectral Density (PSD) for each vehicle is then estimated. It was calculated by Welch algorithm, which is utilized to estimate the power of a signal at different frequencies based on the concept using a periodogram [21]. The PSD is used as the input to the classifier due to the Doppler frequency that contain within the scattered signal. The amplitude for each Doppler frequency follows the radiation pattern of aperture antenna with shapes of vehicle’s silhouette. Thus, the PSD shape is unique to the vehicle’s shape. The next step in the classification system is the amplitude and speed normalization. The amplitude was normalized to the maximum value and speed was normalized based on the relationship of ∆*f = v/L*, where ∆*f* is the FS first null (main lobe width) in the spectrum, where *v* is the speed of vehicle, and *L* is the maximum length of the vehicle [2].

However, the data of spectral signature in PSD is too large, hence Principle Components Analysis (PCA) is used to represent the PSD signature in more valuable information. The data representation in PCA domain is then used for the classification process. The high dimensionality and possibly, highly correlated features from the PSD data is reduced by the PCA. Although PCA will reduce the dimensionality and number of data by exploiting the correlation between the features in PSD, in reality the output will inherently cluster the data to its category. Hence, the output data in the PCA space is used as training and testing vectors prior to the classification algorithm.The paper employed the established KNN algorithm as classifier in the system.

The PCA technique converts high-dimensional data onto a lower-dimensional in a principal component (PC) space. The PCs are arranged so that the amount of variance of the information by each PC is non-increasing. Thus, the first PC carries the highest information of variance in the data and so on. In the PCA algorithm, the input feature vector, *γ* is transform linearly into the new PC space as represented by *Υ* in the equation below [2,20].

(10) $$\Upsilon =Η\cdot {\left(\gamma -g\right)}^{T}$$

where *g* is mean feature vector of the training data and *Η* is a transformation matrix obtained from the training data. If the covariance matrix that was calculated from total training features set is denoted by S, then, the PCA decomposes the covariance matrix S into $S={\mathrm{ULU}}^{T}$. Where L and U is a diagonal N × N matrix of a non-increasing order of the Eigen values and Eigen vectors respectively. The transformation matrix Η is then formed by the Eigenvectors corresponding to the first G highest Eigen value, *Η* = [u1; u2; ...; uG].

The overall classification scheme consists of two stages: the training stage and the classification stage. During the training stage, the entire features vector which is based on the PSD value for all the training set is available. These features of the training sets are correspond to each vehicle category. First step in the training stage is to compute the transformation matrix *H* from the training data by the PCA algorithm. Each training feature vector is then transformed into the PCA-space by using Equation (10). If Q is to represent the vehicle category, then a set of transformed feature vectors {***V***^c^}*_i_* formed for each vehicle category. Feature vectors {***V***^c^}*_i_* is saved and will be used as a reference during the classification process. In the classification stage, the passive FSR system will capture an unknown signal from vehicle crossing the baseline. The new signal is converted into the feature vectors set for individual vehicle. By using the same transformation matrix H, it is then transformed to the feature vector ***V****_j_* and is placed into the PCA space together with Feature vectors {***V***^c^}*_i_*. The final step in classification stage is to apply classifier rule and algorithm to the new signal vehicles’ feature vectors, ***V****_j_* which is unknown. There are many choices of the established classifier method can be applied. The simplest yet reliable classifier method, which is K Nearest Neighbors (KNN) with Euclidean distance, is used. Here three neighbors were chosen to find the smallest distance before category decision was made by the system.

**Figure 9:**
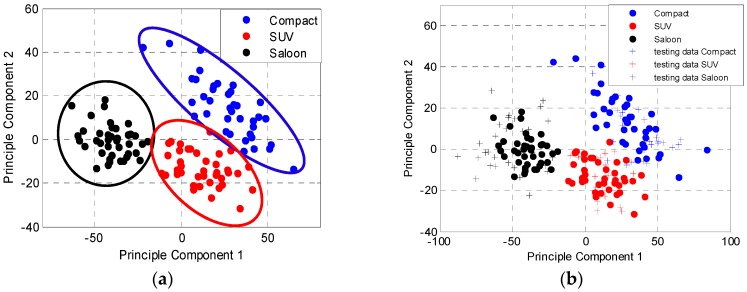
(**a**) Location of training data in PCA space; and (**b**) the spreading of testing data in the database of PC domain.

The training data for each category in the first two principal components in the PC space is illustrated in Figure 9a. The figure shows the data for each vehicle category are positioned at different location in the PC space with slight overlap between the categories. Figure 9b shows the plots of testing data on top of the training data. During the experiment, this paper used the first three principal components for the classification process, which described 91% of the variance of the training data. The full vehicle’s category classification results are presented in Table 3. The results show a high classification performance is obtained with small number of training data. It also shows that the classification system used in passive FSR classifies correctly by 100%, 96% and 92% for Saloon, Small SUV and Compact vehicles, respectively. Although there is slight misclassification by 8% of the Compact as Small SUV, this may be due to the almost similar cross sectional shape between the two categories. The classification system used in the passive FSR system utilize the silhouette shape of the vehicle, which exist in the FS main lobe of the vehicles, therefore the FS main lobe of the Compact would look comparable to the Small SUV except for the different dimensions.

### 3.1. Evaluation of Target’s Baseline Crossing

**Figure 10:**
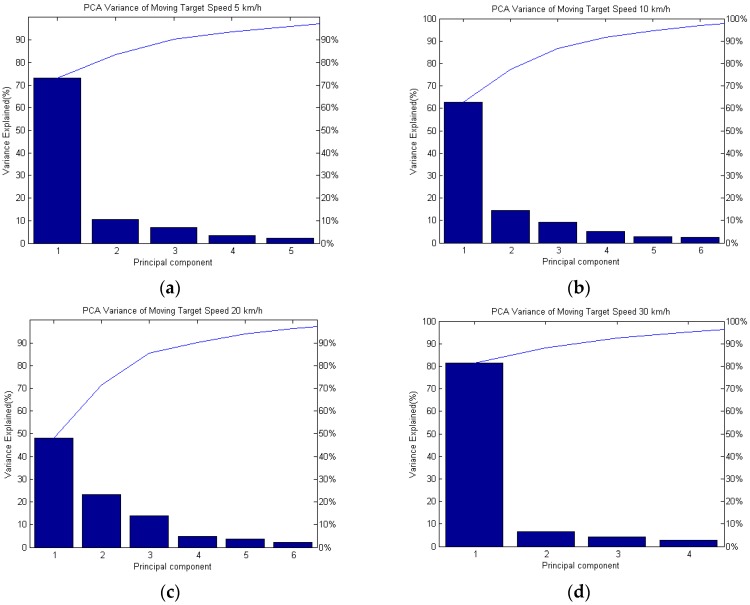
Variance explained of training data for ground moving target with speed of (**a**) 5 km/h; (**b**) 10 km/h; (**c**) 20 km/h and (**d**) 30 km/h in the PC space.

The clustering of the target baseline crossing, *R_R_* (distance between vehicle to the receiver) in passive FSR can be predicted by using the same system as shown in Figure 8. The effect of vehicle’s speed to the overall precision is also analyzed. The variance explained from the output of PCA is utilized for the clustering purposes. In statistics, the explained variation measures the proportion of the mathematical model for the variation of a given dataset. Frequently, variation is quantified as variance, where only then the more specific term ‘variance explained’ can be used [22]. Figure 10 shows the PCA variance explained of the training data for target speeds of 5, 10, 20 and 30 km/h in the PCA space. The results are summarized in Table 4, which highlights the experimental results of the variance explained on the training data. In the experiment, 120 samples were used for each speed of the moving target. Three types of target distance from the passive radar receiver (baseline) were to be recognized by the clustering-based PCA (5, 10 and 20 m). For every type of target’s baseline with the same speed, 40 samples were used. Overall, 480 samples were used for the training data in the principal component space expressed by the first two principal components. Finally, the higher speed of the moving target contributes a finer variance explained, such as 82% of the variance of the training data for 30 km/h, which is the highest among the moving target’s variation of speed, where it is proficiently used for the first principal component. However, for the speeds of 10 km/h and 20 km/h, the variance explained were 63% and 48% respectively as the PCA was built from components such as the sample covariance, which are not statistically robust. This meant that the PCA may be thrown off by outliers and other data pathologies. How seriously this affects the results is specific to the data and application [22].

**Figure 11:**
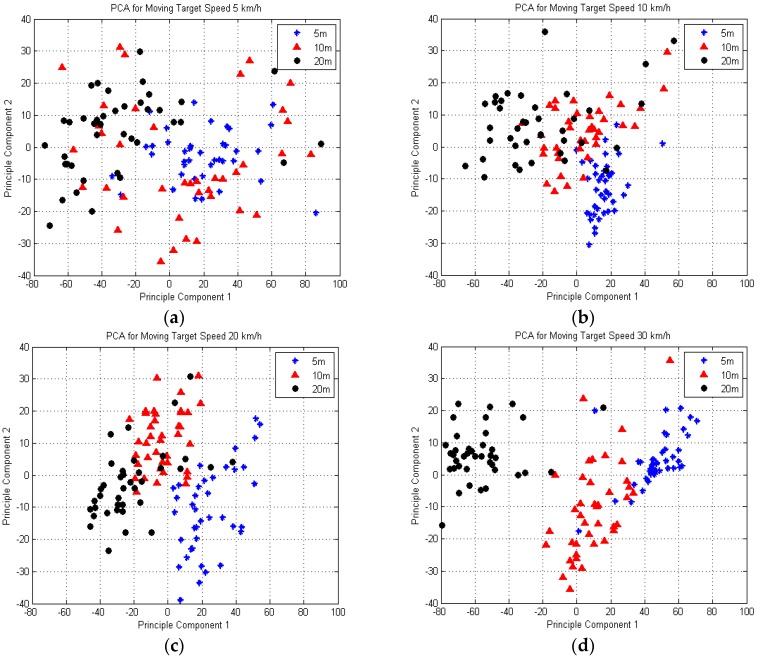
Location of training data for ground moving target with speed of (**a**) 5 km/h; (**b**) 10 km/h; (**c**) 20 km/h and (**d**) 30 km/h in the PCA space.

Figure 11a shows the principal component space for a moving target with speed of 5 km/h. It can be seen that the target’s baseline of 5 m and 10 m were spread all over the graph. However, the baseline of 20 m was almost isolated in the left area of the PCA space while slightly overlapping with the 5 m and 10 m plots. The spread was due to the slow speed of the ground moving target and it stretched out the time domain shape. Figure 11b shows the training data PCA space for 10 km/h, whereby we can almost ascertain that the target’s baseline has its own regions. The blue stars represent the 5 m range of baseline within the range 0 until 40 of the PC1 and −40 until 10 of the PC2. Meanwhile, the red triangles of the 10 m range of baseline take over the middle position of graph. Then, the black dots of 20 m range are localized on the upper-left part of the PCA space while slightly overlaps with the 10 m range. Figure 11c shows a finer resolution for a moving target with a higher speed, 20 km/h. The 5 m range (blue stars) is concentrated on the right side of the PCA space, while the 10 m range (red triangles) is localized in the middle area and the 20 m range (black dots) is confined to the lower-left area of the principal component space. In the case of 30 km/h, which is illustrated in Figure 11d, it seems that each type of target’s baseline has their own area where the black dots (20 m) are localized on the left area, the red triangles (10 m) in the middle area and the blue stars (5 m) are localized on the upper-right area of the PCA space. The results prove that the higher speed of the moving target offers finer clustering on the target’s baseline recognition using PCA analysis. This result also reflects the variance information as mentioned in the previous section, which shows that 82% of variance is achieved at the speed of 30 km/h, thus higher speed indicates better target baseline crossing recognition.

### 3.2. Vehicle Classification Performance at Different Baseline Crossing Range

**Figure 12:**
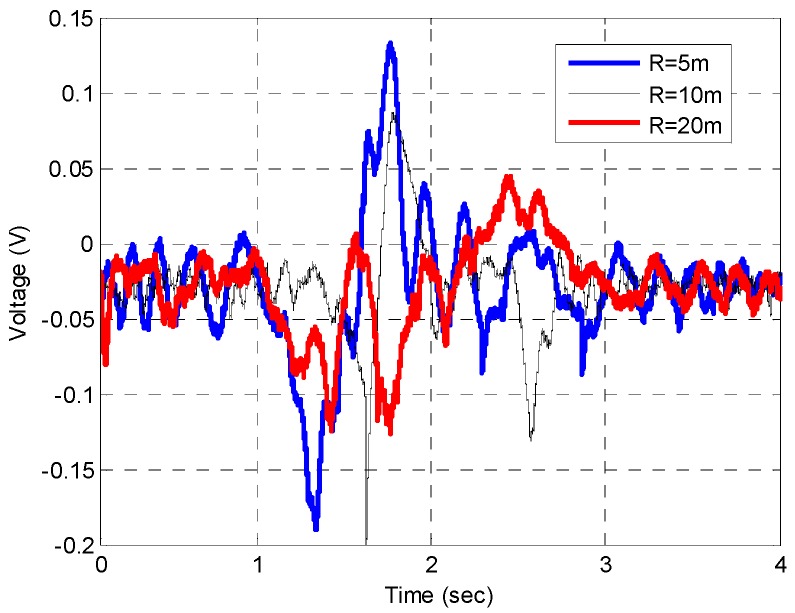
Signal in time at the ADC output for vehicle crossing at different crossing range, *R_R_*.

**Figure 13:**
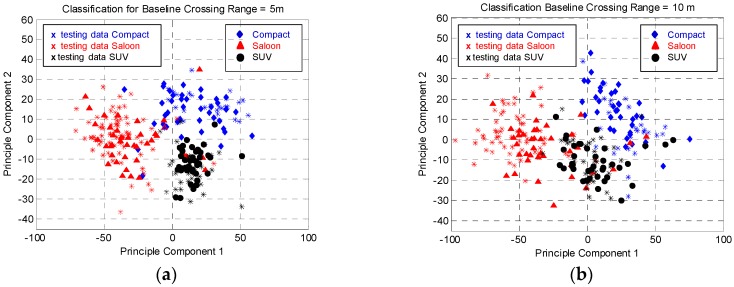

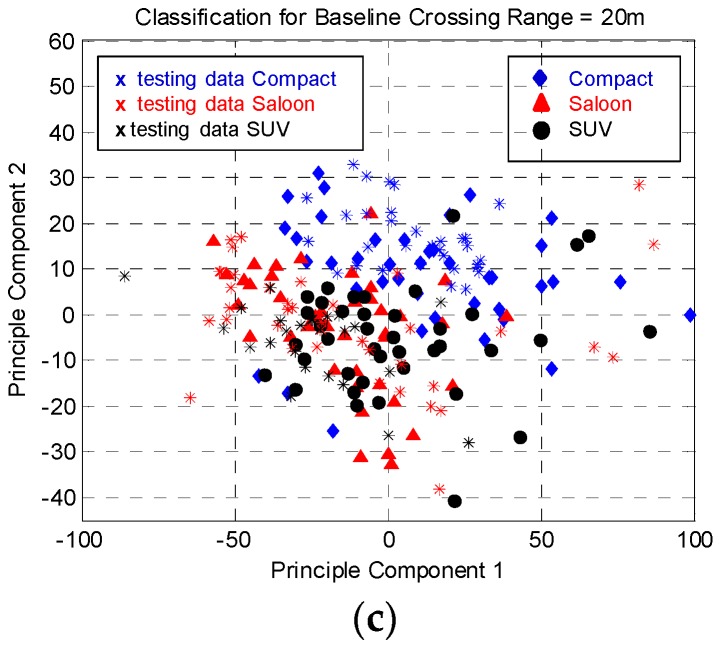
Location of training and testing data for all vehicle categories at different R_R_ in PCA space for (**a**) R = 5 m; (**b**) R = 10 m and (**c**) R = 20 m.

This section analyzes how the different vehicle baseline crossing range, *R_R_* (range from vehicle to the receiver) affect the vehicle classification performance does. It is known that from the FSR theory and as also discussed in Section 2, when a moving target crossing the baseline, the system will gain the enhancement in FS RCS, $\sigma_{f}$ and is given by:

(11) $$\sigma_{f}=4\pi \frac{A^{2}}{\lambda^{2}}$$

where *A* is the physical target area and *λ* the wavelength of the illuminating signal. The amplitude of $\sigma_{f}$ will directly influence signal received at the antenna by:

(12) $$P_{R}=\frac{P_{T}G_{T}G_{R}\lambda^{2}\sigma_{f}}{{\left(4\pi \right)}^{3}R_{T}^{2}R_{R}^{2}}$$

where *P_T_* is the LTE transmitted power from the base station, *G_T_* is the LTE transmitter antenna gain, *G_R_* is the receiver antenna gain. The range from the transmitter to the target is given by *R_T_*. The received signal is highly dependent onthe target size and range between the target and receiver. This data could reflect the signal power at the antenna. The received signal power is inversely proportional to the R_R_ by the inverse square law. However, the range between the target and receiver is restricted in ensuring the FSR condition is met. Figure 12 shows the time domain signal scattered from vehicle in the Saloon category for different *R_R_* with *v* = 10 km/h. Obviously, this signal is the output after all the hardware processing including first RF amplification, amplitude detection via diode, high order frequency filtering by LPF, DC blockage by HPF and low frequency amplifier. It can be seen that the amplitude envelope decreases as the range, *R_R_* increasing. As the system is linear, the envelope does also represent the actual signal power pattern at the front end of the receiver. This signal is then used as the input to the classification system explained in Figure 8. The overall classification performance is tabulated in Table 5 and the location of training and testing data in the PC space is illustrated in Figure 13. As expected, highest classification is observed for *R_R_* = 5 m, which follows the previous indicator explained in Section 3.1. The performance is slightly decreases as the range R_R_ increases which reflect to the quality of the input signal to the classification system. It can be suggested that signal strength plays important role in the classification performance.

## 4. Conclusions

Passive Forward Scatter Radar provides a new emerging area of research that can be utilized for low profile targets as well as ‘Stealth’ targets detection crossing the FS baseline. This paper presents the first experimental result on target detection and classification using passive FSR by exploiting LTE signals as the illuminator of opportunity. The LTE-based passive FSR experiment setup was illustrated in detail. Furthermore, the detection and classification procedures were explained. The selected target’s Doppler spectrum as the target’s feature vectors shows excellent classification result. In addition, the experimental analysis and results prove that the baseline recognition of moving vehicle in passive FSR can be clustered using the Principal Component Analysis. It was found that the speed of the ground moving vehicle influence to the classification result, where higher speed offered finer variance of training data. Moreover, the target’s crossing baseline features by using the target’s power spectrum density had shown good clustering performance. Despite the significant results achieved, an additional pre-processing signal can be included for future analysis to strengthen the target’s baseline crossing recognition in passive FSR. Due to the fact that, this is the first classification analysis of passive FSR to be reported, therefore, future work should also investigate different classification techniques such as, Neural Networks and some Artificial Intelligence can be included in the system.

## Figures and Tables

**Table 1:** Small SUV RCS values for different incident angles, *φ*.

Parameters	Incident Angle, *φ*	
	59°	90°
Bistatic angle, *β* of Max RCS (°)	180.00	180.00
Max RCS magnitude (dBm^2^)	60.04	62.75
Side lobe level (dB)	−31.30	−23.50

**Table 2:** Selected vehicle’s detail for each category used for classification system in passive FSR.

Category	Compact	Saloon	Small SUV
Actual car used during experiment to represent each category	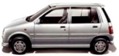	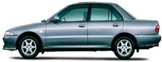	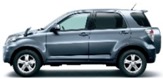
No. of collected data used for classification	70	73	70
Vehicle’s Dimension	*L* = 3395 mm	*L* = 4360 mm	*L* = 4420 mm
	*H* = 1415 mm	*H* = 1385 mm	*H* = 1740 mm

**Table 3:** Vehicle category confusion matrix.

Vehicle-Category	NO.V—For Testing	Automatically Classified as (%)		
		Compact	Saloon	Small SUV
Compact	30	**92**	0	8
Saloon	33	0	**100**	0
Small SUV	30	4	0	**96**

No.V—Number of vehicles for each category in the test data.

**Table 4:** Experimental Results of Variance Training Data.

Target’s Speed (km/h)	5	10	20	30
Variance Explained (%)	73	63	48	82

**Table 5:** Vehicle’s category classification performance at different *R_R_*.

Range, *R_R_* (m)	5			10			20		
Type of Vehicle	Compact	Saloon	SUV	Compact	Saloon	SUV	Compact	Saloon	SUV
Training Data	40	40	40	40	40	40	40	40	40
Testing Data	30	69	33	27	61	35	32	39	25
Classification	30	67	33	21	57	32	30	33	17
Classification (%)	100	97.1	100	77.78	93.44	91.4	93.75	84.62	68.0
**Average Classification (%)**	**99.0**			**87.54**			**82.12**		

## Abbreviations

FSR	Forward Scattering Radar
RCS	Radar Cross Section
LTE	Long-Term Evolution
AF	Ambiguity Function
FSML	Forward Scatter Main Lobe
FS	Forward Scatter
GSM	Global Systems for Mobile
SUV	Sport Utility Vehicle
CST	Computer Simulation Technology
ADC	Analogue to Digital Converter
PCA	Principle Components Analysis
FFT	Fast Fourier Transform
PC	Principle Component
